# Addressing the educational challenges of urban poverty: a case for solution-based research

**DOI:** 10.3389/frma.2023.981837

**Published:** 2023-05-11

**Authors:** Macey Cartwright, Erin O'Callaghan, Sara Stacy, Casey Hord, Heidi Kloos

**Affiliations:** ^1^Department of Psychology, College of Arts and Sciences, University of Cincinnati, Cincinnati, OH, United States; ^2^Criminology, Law and Justice, University of Illinois at Chicago, Chicago, IL, United States; ^3^Department of Psychology, Michigan State University, East Lansing, MI, United States; ^4^Department of Special Education, College of Education, University of Cincinnati, Cincinnati, OH, United States

**Keywords:** out-of-school time, student autonomy, tutors, community-based participatory research, action research, design-based research

## Abstract

**Introduction:**

Math achievement for economically disadvantaged students remains low, despite positive developments in research, pedagogy, and funding. In the current paper, we focused on the research-to-practice divide as possible culprit. Our argument is that urban-poverty schools lack the stability that is necessary to deploy the trusted methodology of hypothesis-testing. Thus, a type of efficacy methodology is needed that could accommodate instability.

**Method:**

We explore the details of such a methodology, building on already existing emancipatory methodologies. Central to the proposed *solution-based research* (SBR) is a commitment to the learning of participating students. This commitment is supplemented with a strength-and-weaknesses analysis to curtail researcher bias. And it is supplemented with an analysis of idiosyncratic factors to determine generalizability. As proof of concept, we tried out SBR to test the efficacy of an afterschool math program.

**Results:**

We found the SBR produced insights about learning opportunities and barrier that would not be known otherwise. At the same time, we found that hypothesis-testing remains superior in establishing generalizability.

**Discussion:**

Our findings call for further work on how to establish generalizability in inherently unstable settings.

## Highlights

- We argue that persistent instability is a pivotal characteristic of urban poverty.
- As initial approximation, a type of efficacy research is proposed, referred to as *solution-based research* (SBR), to accommodate persistent instability.
- We use SBR to test the efficacy of student-guided math practice after school.
- SBR results show that student-guided math practice is engaging and can lead to learning when the right support is in place.
- Our findings indicate that SBR is necessary in addressing educational disparity.

## Introduction

*It's the travel, not the road, that gets you there*.

Matt Hires

Despite numerous positive developments (Kroeger et al., 2012; Schoenfeld, 2016; Neal et al., 2018; Outhwaite et al., 2018), math achievement remains low for students from marginalized communities. For example, the level of math proficiency reported by the National Assessment of Educational Progress [U.S. Department of Education, Institute of Education Sciences, National Center for Education Statistics, and National Assessment of Educational Progress (NAEP), 2019] has remained largely unchanged for children from economically disadvantaged urban communities (e.g., from 18% proficient in 2009 to 17% proficient in 2019). In the current paper, we focus specifically on the research-to-practice divide as a possible culprit for this schism.

### A biased research-to-practice divide

To explain how a research-to-practice divide could be responsible for educational disparity, note that educational success depends on deploying pedagogy that is deemed effective. Hypothesis-testing is the gold standard to determine such efficacy, trusted by journal editors, policy makers, and practitioners. At the same time, it is well-known that hypothesis-testing falls short in reaching marginalized communities (e.g., Brown, 1992; Berliner, 2002; Camilli et al., 2006; Dawson et al., 2006; Balazs and Morello-Frosch, 2013; Sandoval, 2013; Tseng and Nutley, 2014). Building on this work, we highlight a mismatch between the assumptions of hypothesis-testing and the characteristics of urban poverty.

**Figure 1:**
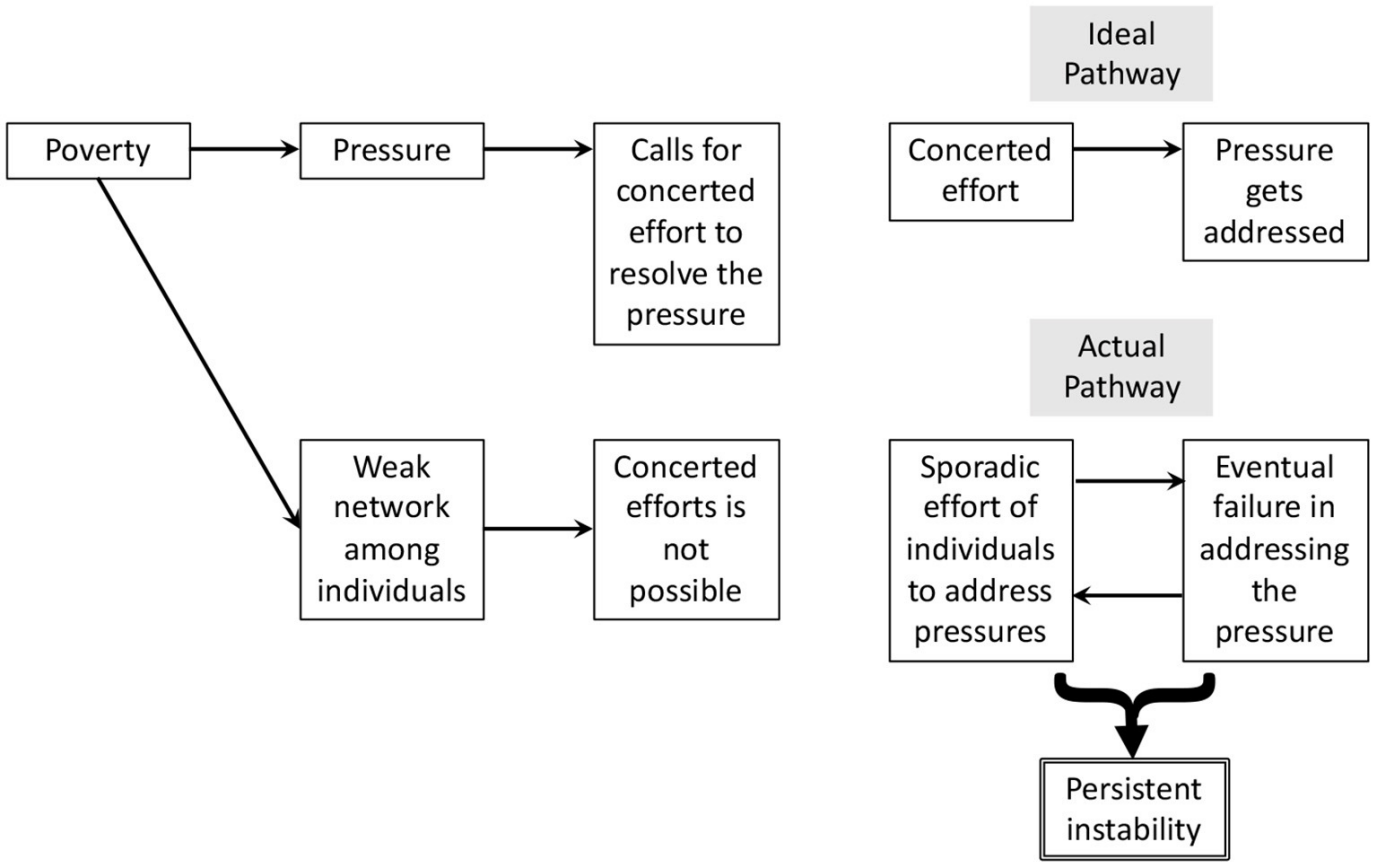
Schematic of the argument that poverty creates persistent instability.

To explain the mismatch, note that the pressures of poverty are serious enough that they cannot be addressed by individuals chipping away at them on their own. No individual can address hunger or crime single-handedly. Instead, a concerted effort of all stakeholders is required. Unfortunately, the constant crisis mode of poverty cuts into the resources needed to grow a cohesive network of individuals working together (cf., Camazine et al., 2003; Evans et al., 2010). Individuals are instead forced to respond to pressures on their own. This yields sporadic efforts that are bound to disappoint. In turn, such ratchet of unsuccessful efforts yields persistent instability (see Figure 1 for a schematic of this logic).

The argument of the poverty-pressure-instability link can also be made at the level of school life. Central here is that urban impoverished schools often fail to reach the educational targets, which earns them the public label of “low-performing,” “failing,” “in need of improvement,” or “on probation,” (Fleischman and Heppen, 2009). Such label comes with the stinging pressure to improve educational outcomes. Yet, it does not come with enough resources to build the cohesiveness among individuals working together to alleviate the pressure. Students, school staff, and parents are therefore forced to operate on their own: A teacher might try a new technology, a parent might organize homework help after school, etc. These efforts are short-lived under the pressure of the massive educational shortfall, leading to persistent instability.

The argument of the poverty-pressure-instability link can even be made at the micro-level of a math classroom. Central here is that the distribution of students' math scores is likely to be strikingly wide, spanning several grade levels (see Appendix A for data in support). Thus, a middle-school math teacher might encounter students who are one, two, and even three grade levels behind, as well as students who are proficient. Such large spread of proficiency creates yet another type of pressure, namely to provide individualized instruction. This pressure is again likely to require a concerted effort of stakeholders (i.e., to organize small-group support). And the resources required to grow such coordination are again missing in poverty. The outcome is a cycle of sporadic efforts and inevitable failures, resulting again in instability.

Incidentally, the hypothesis-testing methodology depends heavily on stability. This is because its logic assumes the presence of a theoretical distribution of scores that represent the status quo (i.e., the “Null” distribution). This distribution can only be estimated when there is enough stability (i.e., the unchanging “instruction as usual”). Yet there is no stable status quo in the ever-changing crisis of poverty, as individuals attempt to address the various pressures of student learning. Put differently, there is no meaningful chance probability in urban poverty, the same way there is no chance probability for the perfect storm (Bernal et al., 1995). For this reason, the central assumption of hypothesis-testing is violated in poverty.

There have indeed been efforts to bring efficacy research to unstable settings (Fenwick et al., 2015). Prominent is the so-called *action research*, a type of efficacy research carried out by practitioners (Stringer, 2008; see also *continuous improvement*; Kaufman and Zahn, 1993; Park et al., 2013). The idea is that practitioners familiar with idiosyncratic constraints could adjust the research protocol accordingly. *Design-based research* (DBR) is yet another efficacy methodology designed to function in potentially unstable settings (Wang and Hannafin, 2005; Kelly et al., 2008). It builds flexibility into the research protocol in order to adjust to idiosyncratic constraints of a setting (Barab and Squire, 2004; Anderson and Shattuck, 2012; Coburn et al., 2013).

While these efforts have expanded the reach of efficacy research into marginalized communities (e.g., Design-Based Research Collective, 2003), both action research and DBR nevertheless rely on a hypothesis-testing protocol. For example, action research counts on practitioners to find a pocket of stability to roll out a hypothesis-testing protocol. And DBR assumes that the idiosyncratic constraints, once known, remain stable for the duration of the research. Thus, these methodologies merely postpone the need of stability, rather than escaping it. Our objective is to develop a type of efficacy research that can produce generalizable insights about an inherently unstable setting.

### A proposal: efficacy research for unstable settings

As initial approximation, we consider the *case-study methodology* as guide (Lipka et al., 2005; Baxter and Jack, 2008; Yin, 2009; Karsenty, 2010). This methodology is intended to study complex phenomena—phenomena that are marked by nonlinear fluctuations (cf., Holland, 2014). The basis for case studies is a collaboration between researchers and participants (Lambert, 2013). A similar collaboration is featured in *community-based participatory research* (CBPR; Israel et al., 1998; Minkler and Wallerstein, 2003; Levine et al., 2005; Ozanne and Anderson, 2010; Wallerstein and Duran, 2010). In CBPR, community members drive the research by having a shared goal between the researchers and community members (see also *activism research, advocacy research, transformative research*).

Building on these approaches, we propose a methodology that is framed by an educational goal shared between researchers and practitioners. Specifically, the so-called *Solution-Based Research* (SBR) is organized by a collaborative effort to improve the learning of students who participate in the research. Following the logic of case-study design and CBPR, such commitment to an educational mission can provide the necessary stability to uncover insights in an inherently unstable system. Note, however, that SBR—unlike case-study design and CBPR—seeks to produce efficacy results about a pedagogical intervention.

One could argue that a close involvement on the ground could elicit researcher bias (cf., Malterud, 2001; Mann, 2003). In response, we consulted the methodology of *evaluation research* as guide. This type of methodology is concerned with the effectiveness of a program without the use of hypothesis-testing. Insights about a program are instead obtained *via* a thoughtful analysis of the program's strengths and weaknesses (Patton, 1990; Quinn, 2002; Narayanasamy, 2009; Ghazinoory et al., 2011). The idea is that, while researcher insights are subjective, the process of highlighting both strengths and weaknesses of an intervention forces a mindset of discovery. In turn, such mindset creates a balanced approach that could keep biases in check. We propose that SBR employs a strengths-and-weaknesses analysis too.

In addition to curtailing researcher bias, efficacy research also needs a strategy by which to determine whether findings can be generalized. In hypothesis-testing, generalizability is accomplished by estimating the chance probability of descriptive results. Given the absence of such chance distribution, we propose to accomplish generalizability *via* a systematic analysis of idiosyncratic factors vis-à-vis existing literature. This same idea is used in both case-study research and evaluation research to obtain transferable insights. Thus, there is precedence for the claim that an open-mined consideration can address the question of generalizability.

Taken together, SBR has three central features (see Table 1 for an overview). First, SBR adds to the stability of educational settings by supporting the learning of students. This requires a partnership with the educational setting, namely to identify the needs and resources relevant to the intervention. Second, SBR involves a critical analysis of strengths and weaknesses of the intervention, namely to curtail researcher bias. And third, SBR involves a generalizability analysis to determine the degree to which observed trends might hold up in the larger population. In what follows, we illustrate SBR with a concrete example.

**Table 1:** Overview of the proposed SBR methodology.

**Goals of SBR**	**Central features**	**Parts of SBR**	**Related methodologies**
Create stability	Support the learning of students	• Build connections with the community partner • Identify needs and resources • Adapt the intervention • Identify measuring tools that support learning	Community-based participatory research Action research Needs assessment Design-based research Case-study design
Minimize researcher bias	Critically analyze strengths and weaknesses	• To what extent was the intervention engaging? • To what extent did the intervention lead to learning?	Evaluation research Continuous improvement
Obtain generalizable insights	Explore idiosyncratic factors vis-à-vis published findings	• To what extent are selective findings generalizable?	Evaluation research Case-study design

## SRB in action: an efficacy study on an afterschool math program

### Introduction

As example case, we sought to investigate the benefits of math practice, the idea being that students gain competence when they solve math problems at an appropriate difficulty level (Frye et al., 2013; Jansen et al., 2013; Haelermans and Ghysels, 2017). We were specifically interested in a type of math practice that gives students agency about what to practice. Student autonomy is known to act as a powerful motivator, likely to increase engagement (Pink, 2009; León et al., 2015). In order to make such individualized practice feasible, we used technology in combination with adult facilitators (Karsenty, 2010; Bayer et al., 2015; Stacy et al., 2017). Our study sought to explore: To what extent is the proposed student-guided math practice effective?

### Method

Table 2 provides an overview of the method used for this study. Importantly, students who participate in SBR are not research participants *per se*. This is because all of the activities students complete are designed for educational purposes. Thus, SRB method does not have a *Participants* section.

**Table 2:** Overview of the example SBR method.

**Setting**	**Intervention**	**Measuring tools**
• Middle-school students • Remedial math • Afterschool space	• Students autonomy • IXL Technology • College-student facilitators	• Field notes • Math assessments • Surveys on math attitudes • IXL analytics • Feedback from facilitators • Feedback from school staff

#### Setting

The partnering school was a large urban high school that covered Grades 7–12 (~150 students per grade level). At least 90% of students were classified as economically disadvantaged at the time the study took place. The relevant school metrics were predictably troubling: 84% of students scored below proficient on state-wide math assessments, chronic absenteeism was 26%, and high-school dropout was 27%. For 7th-graders specifically, only 20% of students passed the state-wide math test in the year before the research was carried out.

At the same time, the school had extensive collaborations with local organizations, which has led to numerous initiatives (e.g., STEM exposure, career explorations, support in health and wellbeing). It also attracted funding through the 21st Century Community Learning Centers (21st CCLC) funding mechanism to provide academic enrichment opportunities during out-of-school time (James-Burdumy et al., 2005; Leos-Urbel, 2015; Ward et al., 2015). The partnership with researchers was created to design an afterschool math enrichment program. The decision was made to carry out an afterschool math program for 7th and 8th grade students.

#### Intervention

The intervention was a student led afterschool math practice program, blending technology and the human element. Regarding technology, we opted for the math practice app *IXL* (IXL Learning, 2016). This app provides access to a comprehensive library of practice sets from all K-12 Common Core topics, organized by grade level and math topic. Regarding the human element, we opted for college-student volunteers to act as facilitators. There was no requirement for facilitators to be math proficient beyond general college readiness. Their task was to help students choose practice sets that were neither too easy nor too difficult for them. For example, should a student visibly struggle with a practice set, facilitators had to guide the student toward an easier practice set.

Following the requirement of the 21st CCLC funding, the math-practice program was offered after school. Two sessions were offered per week to accommodate the schedule of as many students as possible (one hour per session). An extensive reward system was in place to encourage student attendance (e.g., raffle tickets, tokens to be redeemed, opportunities for students to make up a demerit). There were also information booths at school events to let parents know about this opportunity, as well as reminder calls to parents the night before a session. A meal was available to students prior to each session.

Students accessed *IXL* on a provided tablet or laptop that connected to the school's Wi-Fi. To encourage a prompt start, a session began with a “warm-up” practice set that earned students a treat. After the warm-up, students were free to decide what practice set to work on. When they solved a problem correctly, an encouraging statement appeared on the app (e.g., “good job”), after which the next math problem was loaded. When students entered an incorrect answer, an explanation appeared and students had to press a button to move onto the next math problem. A “Smart Score” visible on the screen tracked the student's performance within each practice set. This value increased with every correct answer and decreased with every incorrect answer, culminating in a score of 100.

#### Measuring tools

*Field notes*. We developed an observation protocol to capture the behavior of students and facilitators during a session. For students, positive indicators were to actively work on *IXL* or seek help from a facilitator. Negative indicators were to be distracted or frustrated, or to quit the math practice altogether. For facilitators, positive indicators were to be attentive to students' work or provide encouragement to a frustrated or inactive student. Negative indicators were to be distracted (e.g., chatting with other adults) or to give lengthy explanations to students. Field notes also served as an attendance record.

*Student math proficiency*. We gauged students' math proficiency at the onset of the program. Two subscales of the Woodcock-Johnson test battery (Version IV) were used for this purpose: math fluency and calculation competence. The math fluency subscale is a 3-min test of simple one-digit operations (addition, subtraction, multiplication). The calculation-competence subscale is an untimed test of arithmetic, fractions, algebra, etc. Both subscales return the student's grade equivalence (GE) score. We also gauged students' learning *via* the analytics obtained by *IXL* (e.g., duration of practice, type of practice, error reports).

*Student attitudes toward math*. Two measures were developed to capture students' attitudes toward math, one administered at the onset of the program, and one administered after each session. Appendix B shows the intake survey: It focused on whether students like math (e.g., “Is there something that you like about math?”), their beliefs about math competence (e.g., “How good do you think you are at math?”), and their coping skills when encountering a difficult math problem (e.g., “Do you let someone help you?”). After each session, we also asked: “What did your face look like what it was time for math today?” There were five answer options ranging from “not nervous” to “very, very nervous.”

*Feedback from facilitators and school staff*. A program-satisfaction survey was administered to facilitators at the end of each semester. Anonymously, facilitators were asked to describe positive and negative aspects of the program. Facilitators were also interviewed after each session to capture possible opportunities and barriers to learning. Teachers and school administrators were interviewed throughout the year about the program and to explore potential solutions to issues we encountered. They were also interviewed at the end of the year to capture perceived strengths and weaknesses of the program. All members of the school staff involved with the program were available for at least three interviews over the course of the year.

### Results

With permission from the school's administrators, de-identified data were released for research purposes. These data were then mined for two reasons: to determine the strengths and weaknesses of the student-guided math practice, and to determine the extent to which the observed findings could yield generalizable conclusions. For the current study, we were specifically interested in whether the intervention was engaging and led to learning.

#### Analysis of strengths and weaknesses

Our approach was to generate an initial list of strengths and weaknesses regarding student engagement and learning. This list was then checked against available evidence and modified iteratively until the set of strengths and weaknesses was balanced in quantity and quality. Below, we describe the finalized list of items (see Table 3).

**Table 3:** Outcome of the strengths-and-weaknesses analysis.

**To what extent was student-guided math practice engaging?**		**Evidence**
Strengths	• Students started promptly and sustained interest • Each aspect of the program was well-received	• Field notes • Facilitator feedback • School staff feedback • Field notes
Weaknesses	• The program generated low spontaneous attendance • There was high uncertainty due to variability in student attendance	• Field notes
**To what extent did student-guided math practice lead to learning?**		
Strengths	• Students improved in many math skills they practiced	• IXL analytics • Math assessments • School staff feedback
Weaknesses	• Errors often interrupted learning	• Field notes • Facilitator feedback

**Figure 2:**
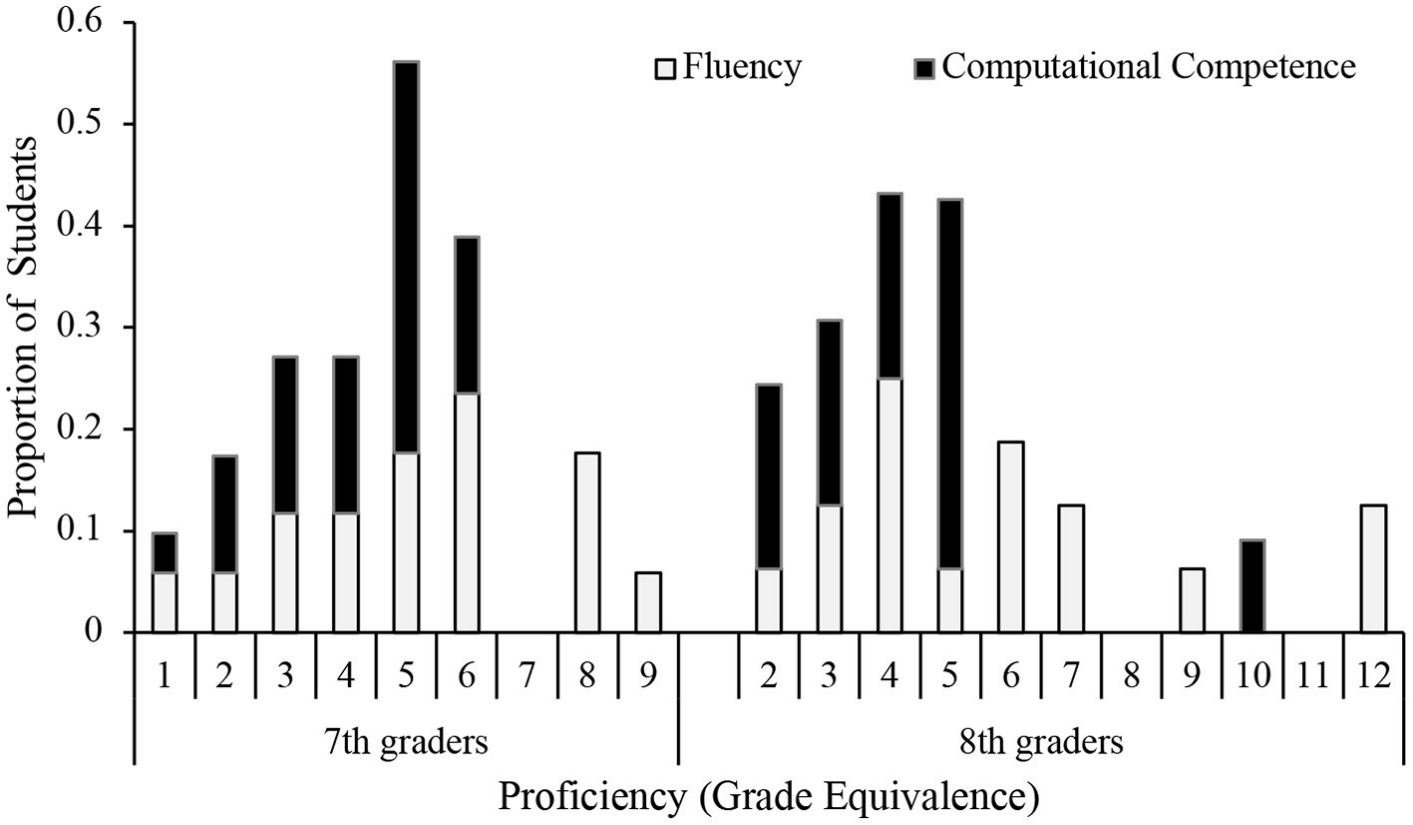
Proficiency scores expressed as proportion of students scoring at a certain grade level (*N* = 45 7th-graders; *N* = 48 8th-graders). There was a large variability in proficiency: While some students scored at the 1st- and 2nd-grade proficiency level, other students scored above their actual grade level. Average proficiency in math fluency was close to grade level for 7th grade students (*M* = 6.97, SD = 2.32), but below grade level for 8th grade students (*M* = 6.50, *SD* = 2.79). Average proficiency in calculation competence was substantially below grade level for both 7th (*M* = 4.55, SD = 1.36) and 8th grade students (*M* = 5.25, SD = 2.14). There was a significant correlation between fluency and calculation competence, *r*(24) = 0.70, *p* < 0.05.

*Was self-guided math practice engaging?* Our observations showed that students were eager to start their practice. They also were willing to practice math for the duration of the full hour. This applied to a large variety of students, independently of their initial proficiency (see Figure 2 for a distribution of proficiency scores), and independently of their attitudes toward math (27% reported to be “not so good” in math, 68% reported that they dislike math). Even students who attended involuntarily were compelled to practice math. In fact, some students asked to continue practicing after the hour was up. Teachers and visitors to the classroom commented on the positive energy and student focus. To quote a facilitator (survey): “with enough [adult] support, it worked like a well-oiled machine.”

Comments from students and facilitators indicated that each of the program's aspects was well-received (student autonomy, technology, facilitators): Students were appreciative of being given a choice about what to practice, and there was no obvious misuse of this privilege. Students also found the *IXL* app exceedingly easy to navigate, needing virtually no assistance with using the app correctly. And students connected well with the facilitators, especially when there was consistency in student-facilitator pairing. Students clearly enjoyed the interactions with the facilitators they were familiar with, and they were visibly upset when they could not work with “their” facilitator.

**Figure 3:**
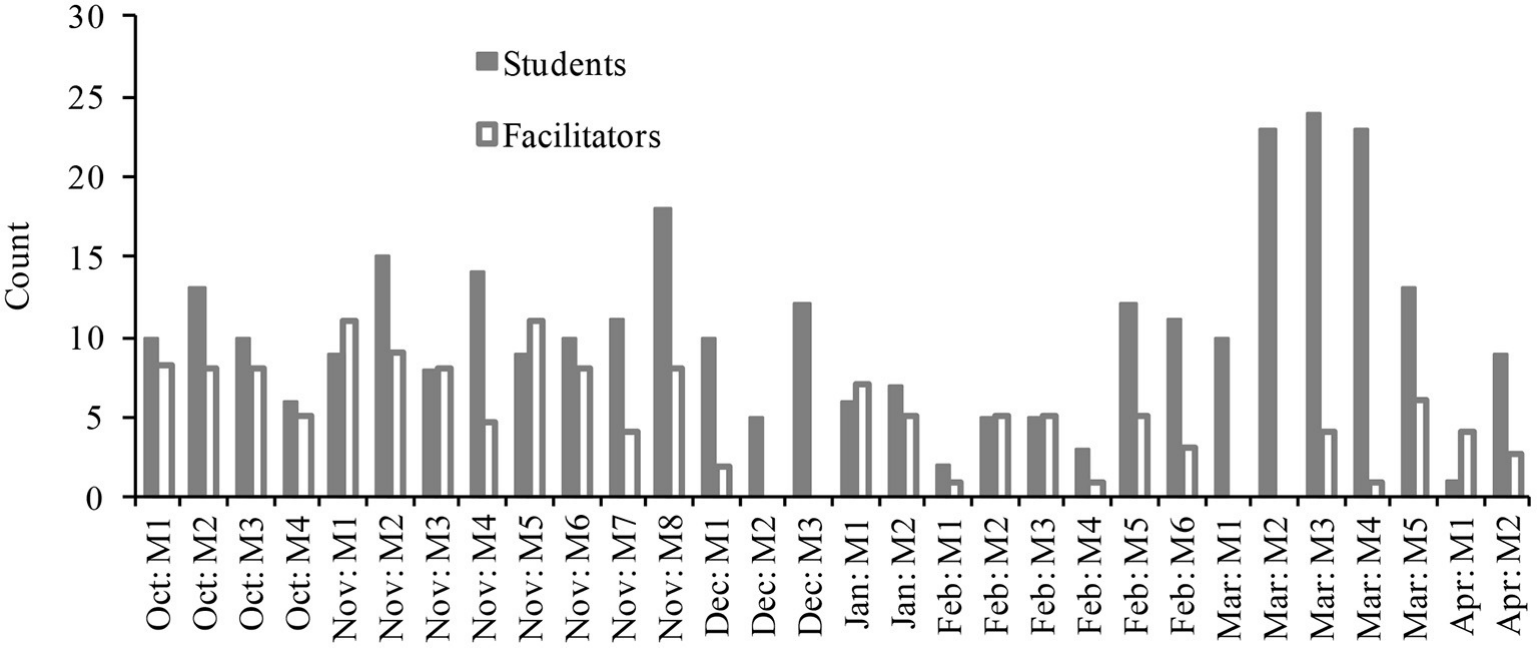
Attendance of students and facilitators across the year, plotted for each meeting (M).

Yet, student engagement did not yield reliable attendance. Considerable efforts were undertaken by school staff to make the afterschool space work. In some cases, coaches even mandated attendance of their players. These efforts led to sporadic increases in attendance (see Figure 3). However, they had little effect on students' voluntary attendance (e.g., only 19% of *N* = 93 students attended regularly). In fact, they had the unintended consequence of leading to sharp fluctuations in attendance (from 1 to 24 students per session). In turn, the student-facilitator ratio fluctuated as well, ranging from 1:1 to 4:1. This created uncertainty for both students and facilitators (e.g., routines could not solidify; excessive time had to be spent explaining the program to new-comers). Facilitators also reported on being overwhelmed at times, as it was difficult to manage large groups of students.

*Did student-guided math practice lead to learning?* We found that students clearly benefited from math practice. *IXL* analytics showed that students who participated in five or more sessions (*N* = 18) improved in division (80% of students), operations with integers (75% of students), multiplication (50% of students) and equations (27% of students). The comments of students and teachers further substantiated these findings. For example, an 8^th^-grader explained to a teacher visiting the program, “This is why I was so good at slopes in class today—I've been practicing.” Neither initial math proficiency nor math attitude appeared related to degree of learning.

At the same time, learning was interrupted when students made errors. Observations showed that students were frustrated over errors—even attempting to abandon math practice all together (e.g., switching off the tablet, laying their head on the desk). According to facilitator input, students also failed to learn from their errors: Students showed little interest in the explanations of errors offered by IXL, and they continued to make the same mistakes when facilitators took it upon themselves to explain an error. Switching to an easier practice set was far from straightforward: Observations and student responses indicated that students had little insight about their own gaps in math proficiency, making it difficult to choose appropriately challenging practice sets.

#### Generalizability analysis

To what extent could our descriptive findings apply generally? Our approach was to first narrow down the list of findings of interest. For each chosen finding, we then mined the literature to determine whether published results align with it. Next, we considered idiosyncratic factors that could account for the finding. Based on this information, we then speculated on whether the finding could be generalized (see Table 4 for an overview of the outcome of this analysis).

**Table 4:** Outcome of the generalizability analysis.

**Descriptive results**	**Existing literature**	**Idiosyncratic factors**	**Generalizability**
• Students were motivated to practice math	Extensive	Engagement was strong across different students	Likely
• Attendance fluctuated unpredictably	Unclear	There were sporadic efforts to increase attendance	Unclear
• Students improved on what they practiced	Extensive	Not everybody improved in all areas	Unclear
• Errors interrupted learning	Some	IXL practice sets were not organized by difficulty level	Limited

*Is student engagement likely to generalize?* Descriptively, we found that all three elements of the student-guided math practice were motivating to students: Students appreciated being given a choice, they enjoyed using the *IXL* app, and they bonded with the facilitators. These findings are strongly aligned with existing literature (Baker et al., 1996; Slavin et al., 2009; George, 2012; Leh and Jitendra, 2013). Regarding idiosyncratic factors, we found that student engagement was high, independently of their attitudes, math proficiency, or the reason for attending the program. Therefore, it is likely that the combination of autonomy, technology, and facilitators is motivating to students more generally.

*Is the fluctuation in attendance likely to generalize?* Descriptively, we could not establish reliable attendance after school, despite extensive efforts to do so. This finding was surprising, given that the afterschool space is a popular choice for learning (Lauer et al., 2006; Apsler, 2009; Afterschool Alliance, 2014). It is possible, therefore, that our finding stems from idiosyncratic factors, whether of the program, the school culture, or student life (e.g., the stigma of remedial math). At the same time, the fluctuations in student attendance mirror the instability characteristic of urban poverty. Like education itself, afterschool attendance too might require the concerted effort of an underdeveloped network that results in a cacophony of well-meaning but unorganized efforts.

*Is student learning likely to generalize?* Descriptively, we found that students improved in many of the skills they practiced. This finding is unsurprising given the general understanding that practice leads to learning (Jonides, 2004; Woodward et al., 2012). At the same time, learning was not uniform. For example, practicing division problems led to far more improvement than practicing equation problems. Exploring the published literature, we had difficulty finding research that could shed light on such nuanced effect of math practice. In fact, we were surprised to find relatively little research on math practice, given that math practice is common in students' life (e.g., homework). Thus, we conclude that it remains unclear whether our practice results can generalize.

*Is the effect of errors likely to generalize?* Descriptively, we found that errors interrupted learning, whether because students got frustrated, because they had difficulty learning from their mistakes, or because they lacked strategic knowledge about what to practice next. There is indeed research on how pressure leads to strong emotional responses, which, in turn, curtails decision-making (Pearman, 2017). At the same time, there are numerous idiosyncratic factors that could account for our findings. For example, facilitators sometimes offered a reward for finishing a practice set, inadvertently making errors relevant. And *IXL* is not designed to make difficulty levels obvious, thus failing to guide students' choices. Note also that errors were far less disruptive when the student-facilitator ratio was small. Therefore, we conclude that these findings generalize when settings lack the necessary support.

### Summary on student-guided math practice

Our findings suggest that student-guided math practice is an effective pedagogical tool to promote math practice among students from urban impoverished communities. We also found evidence for learning, at least for some math topics. At the same time, afterschool attendance fluctuated sharply and student errors often led to frustrations and inefficiencies that interrupted learning. Further research is needed to investigate whether afterschool attendance is a broader concern in urban-poverty schools. Research is also needed to explore ways to support students' math practice in ways that minimizes frustrations and inefficiencies.

## General discussion

Our starting point was the argument that education in urban impoverished neighborhoods suffers from a research-to-practice divide because it lacks the stability that is needed for hypothesis-testing. Solution-based research was proposed in response, namely to produce efficacy results in settings marked by persistent instability. SBR involves (1) a commitment to the learning of participating students, (2) an analysis of both strengths and weaknesses of the intervention, and (3) an analysis of idiosyncratic factors vis-à-vis existing literature to determine generalizability. In light of our study, we discuss each of these aspects.

Consider first the commitment to the learning of participating students. We found that this aspect of SBR worked remarkably well to get research off the ground. Teachers and school staff were eager to help, and they made available needed resources, space, and information. The explicit goal of supporting students also generated a lot of good-will to make the program a success, even among individuals who were not directly related to the project (e.g., sports coaches). The flexibility in the research protocol might have empowered individuals to get involved, creating a collaborative atmosphere among teachers, school staff, and parents. Thus, the educational goal of SBR—put in place to accommodate instability—also forged an educational community. Such community building is likely to be at the heart of addressing the pressures of poverty.

Next, consider SBR's critical analysis of the intervention's strengths and weaknesses. Here too, we had encouraging findings. Notably, the exploratory search for positives and negatives of the intervention brought out diverse points of views of researchers and practitioners. These interactions led to a learning experience for individuals who approached math education from very different vantage points. Thus, preconceived notions had to be revised, in effect neutralizing potential biases. A nuanced picture about opportunities and barriers to learning emerged instead. For example, discussions with practitioners helped highlight the plight of students averse to math, as well as the enormous challenge of math teachers working in large-class settings.

At the same time, we encountered some shortcomings of the strengths-and-weaknesses analysis, especially when it comes to deciding on what findings to consider. In the current study, we focused on the intervention's effects on student engagement and learning. However, this was not the only focus we tried out. In fact, we went through several iteration of strengths and weaknesses, examining separate aspects of the program and its outcomes (e.g., effect on student-facilitator relations). Arguably, such unconstrained process slows down the analysis.

Finally, consider SBR's generalizability analysis. Here we had to select pertinent findings, mine the literature for confirmation, and then consider idiosyncratic factors that could be at play. Needless to say, it was unfeasible to carry out systematic literature reviews on issues that arose. It was also impossible to consider all possible idiosyncratic factors without full information about students' lives. Instead, we found ourselves relying on circumstantial information. Thus, we had to enlist our intuition when deriving claims about generalizability. By comparison, neither circumstantial information nor intuitions are needed for hypothesis-testing. One merely has to enter the data and then read off the result of whether the criterion of generalizability is met.

Seeing that a hypothesis-testing protocol remains superior on the question of generalizability, one could argue that impoverished communities could forego their own efficacy research and rely instead on findings obtained from affluent schools. This might be the logic behind the requirement that low-performing schools should employ evidence-based interventions without concern about where the evidence was obtained (e.g., No Child Left Behind, 2002; Every Student Success Act, 2015). Indeed, the efficacy results compiled for practitioners rarely list the demographics of participants [e.g., see U.S. Department of Education, Institute of Education Sciences, National Center for Education Evaluation and Regional Assistance, What Works Clearinghouse (2019), edreports.org, achievethecore.org]. Our findings advise against this logic: The educational landscape of urban poverty seems sufficiently unique to warrant its own efficacy research (cf., Anna-Karenina principle of “All unhappy families are different”; Diamond, 1997). For this reason, SBR might be indispensable.

## Conclusions

The quote at the top of our paper anticipates the difference between efficacy research (the travel) and the well-trodden path of hypothesis-testing (the road). Under ideal circumstances, efficacy research takes place on the established road of hypothesis-testing. Urban poverty is not such an ideal circumstance, however. Its persistent instability runs counter to the assumptions of hypothesis-testing. In response, we explored a type of efficacy research that can accommodate persistent instability, building on already existing methodologies. Though the proposed solution-based research does not have an established rule book, our findings highlight several reasons why it might be worth continuing the travel. The hope is that such travel might eventually make a road.

## Data availability statement

The raw data supporting the conclusions of this article will be made available by the authors, without undue reservation.

## Ethics statement

The studies involving human participants were reviewed and approved by Institutional Review Board of the University of Cincinnati (Protocol # 2014-4138). The patients/participants provided their written informed consent to participate in this study.

## Author contributions

All authors listed have made a substantial, direct, and intellectual contribution to the work and approved it for publication.

## Appendix A

**Table A1:** Math performance of 4^th^- and 8^th^-grade students in 2017, reported in NAEP, 2019.

	**Raw scores**		**Proportions of students**				
	**Average**	***CoV***	**Below Basic**	**Basic**	**Proficient**	**Advanced**	***CoV***
**4^th^ grade**							
Urban NSLP eligible	225	0.13	35	43	20	2	0.62
Suburban NSLP ineligible	255	0.11	8	31	45	16	0.49
**8^th^ grade**							
Urban NSLP eligible	265	0.14	48	35	14	3	0.74
Suburban NSLP ineligible	300	0.12	15	33	33	19	0.60

Students' eligibility for the National School Lunch Program (NSLP) is taken as proxy for income (i.e., low-income children are eligible to receive reduced-price or free meals at school). We cross-tabulated school location (urban vs. suburban) with NSLP eligibility. As expected, there is a sizable difference in math achievement between the two groups (whether we considered average scores or the proportion of students scoring below-basic proficiency levels, *ps* < 0.001). We also calculated two coefficients of variation (*CoV*)—one for raw scores and one for students' competence level—to capture the range in scores. Notably, the distribution of scores is far wider for urban low-income students than for their suburban counterparts.

## Appendix B

**Math intake survey for students**

Attitudes toward math:

1. Is there something that you like about math? [If yes] What do you like about math?
2. Is there something that you don't like about math? [If yes] What is it you don't like about math?
3. What does your face look like when it is time for math?
4. What does your face look like when math is over?
5. What would your face look like if you were to find out you never had to do math again?
6. Do you think you would like to have a job that has a lot of math?
  [If yes] what would you like about a job that involves a lot of math? It's OK if you are not sure. Just say “I'm not sure.”
  [If no] what would you not like about a job that involves a lot of math?

Perceived competence:

1. How many people do you think are good at math? Are *all* people good at math, *many* people, *some* people, or *only a few* people?
2. How good do you think you are at math? Are you *very good, good, okay*, or *not so good*?
3. How good do you think boys are at math? Are they *very good, good, okay*, or *not so good*?
4. How good do you think girls are at math? Are they *very good, good, okay*, or *not so good*?

Coping strategies:

Imagine you have a difficult math problem on homework. Did that ever happen to you? What do you remember about it? Can you tell me about it? What do you do when you have a problem like what you just told me about?

- Do you say to yourself: “it isn't so serious”?
- Do you do something to take your mind off it?
- Do you wonder what to do?
- Do you say to yourself: “I know I can solve the problem”?
- Do you ask somebody what to do?
- Do you try to get out of having to do it?
- Do you keep worrying and thinking about the problem?
- Do you want to give up?
- Do you get grumpy?
- Do you make a plan to fix the problem?
- Do you let someone help you?
- Do you try to figure it out and solve the problem?
- Do you get mad at the teacher?
- Do you pretend you don't care?
- Do you say to yourself: “I know I can fix this”?
